# Current Status of Monoclonal Antibodies-Based Therapies in Castration-Resistant Prostate Cancer: A Systematic Review and Meta-Analysis of Clinical Trials

**DOI:** 10.7759/cureus.22942

**Published:** 2022-03-07

**Authors:** Talha Azam Tarrar, Muhammad Yasir Anwar, Muhammad Ashar Ali, Memoona Saeed, Sana Rehman, Shammas F Bajwa, Tooba Ayub, Haleema Javid, Rimsha Ali, Alaa Irshad, Wajeeha Aiman

**Affiliations:** 1 Internal Medicine, Nottingham University Hospitals NHS Trust, Nottingham, GBR; 2 Internal Medicine, BronxCare Health System, Bronx, USA; 3 Internal Medicine, Beth Israel Deaconess Medical Center, Harvard Medical School, Boston, USA; 4 Internal Medicine, Mayo Hospital, Lahore, PAK; 5 Internal Medicine, Shaikh Khalifa Bin Zayed Al-Nahyan Medical and Dental College, Lahore, PAK; 6 Internal Medicine, AdventHealth Orlando, Orlando, USA; 7 Internal Medicine, MacNeal Hospital, Berwyn, USA; 8 Internal Medicine, Rawalpindi Medical University and Allied Hospitals, Rawalpindi, PAK; 9 Internal Medicine, Rawalpindi Medical University, Rawalpindi, PAK; 10 Internal Medicine, Allama Iqbal Medical College, Lahore, PAK

**Keywords:** castration-resistant prostate cancer, meta-analysis, systematic review, checkpoint inhibitors, monoclonal antibodies, prostate cancer

## Abstract

Background

Multiple patients with prostate cancer become resistant to castration therapies, which is termed castration-resistant prostate cancer (CRPC).

Purpose

The purpose of this review is to assess the status of efficacy (≥50% decline in prostate-specific antigen (PSA), progression-free survival (PFS), and overall survival (OS)) and safety (grade 3-4 adverse effects) of monoclonal antibodies in CRPC.

Data source

We searched databases including PubMed, Embase, Cochrane, Web of Science, and ClinicalTrials.gov.

Results

Hazard ratios of PFS and OS were 0.77 (95% CI = 0.69-0.87, I^2^ = 53%) and 0.98 (95% CI = 0.86-1.11, I^2^ = 40%), respectively, in the favor of monoclonal antibodies as compared to placebo. Risk ratio (RR) of >50% decline in PSA was 1.99 (95% CI = 0.97-4.08, I^2^ = 53%) in favor of monoclonal antibodies. Pooled incidence of >50% decline in PSA levels was 15% (95% CI = 0.1-0.23, I^2^ = 83%), 29% (95% CI = 0.14-0.51, I^2^ = 93%), 63% (95% CI = 0.49-0.76, I^2^ = 77%), and 88% (95% CI = 0.81-0.93, I^2^ = 0%) in single, two, three, and four-drug regimens, respectively.

Conclusion

Monoclonal antibodies are well tolerated and showed better PFS as compared to placebo. However, OS was only improved with ipilimumab. Denosumab delayed skeletal-related adverse events as compared to zoledronic acid. More multicenter double-blind clinical trials may be needed to confirm these results.

## Introduction

Prostate cancer is the second most common cause of cancer deaths in men after lung cancer in the United States with both aggressive and slow-growing types identified. More than 20% of the newly diagnosed cases of cancer are prostate cancer [1]. The new cases and estimated deaths for prostate cancer reported in the US in 2019 were 174,650 and 31,620, respectively, with an increase in the trend seen in 2020 with 191,930 new cases and 33,330 estimated deaths [1,2]. Globally, 1,276,106 new cases were estimated in 2018. Developed countries have higher incidence probably due to better use of diagnostic testing [3].

The various modalities that continue to be the mainstay of treatment for prostate cancer are surgical (prostatectomy), hormonal (gonadotropin‐releasing hormone agonist or antagonist, androgen deprivation), and radiation (external beam radiotherapy, brachytherapy) [4-6]. However, surgical/chemical castration is required for most patients with metastatic disease. The progression of the carcinoma with or without metastasis despite castration therapy (androgen deprivation therapy) is termed as castrate-resistant or hormone-resistant cancer and is characterized by rising prostate-specific antigen (PSA) levels with castrate range of testosterone (<50 ng/dl or <1.7 nmol/l) [6-9].

Chemotherapy agents including taxanes, bisphosphonates, immunotherapy agents, and poly (ADP-ribose) polymerase-1 inhibitors have shown anti-tumor activity in patients with castration-resistant prostate cancer (CRPC). Taxane with prednisone is the most common treatment used for CRPC. Despite these treatment options, the prognosis and quality of life of these patients are very poor. There is still room for more combination therapies for the treatment of CRPC, especially for patients who do not tolerate and/or are refractory to first-line therapies [10-13].

In recent years, monoclonal antibodies have shown promising results in clinical trials. Monoclonal antibodies have been evaluated for their efficacy in CRPC due to their targeted action on various tumor factors that help control cancer progression [4]. The most common antibodies studied include bevacizumab (anti-vascular endothelial growth factor (VEGF)), which decreases angiogenesis and improves vessel penetration of cytotoxic agents like taxanes when used in combination [10,11]. Cixutumumab and ramucirumab act against insulin-like growth factor-1 receptor (IGF-1R)/vascular endothelial growth factor receptor (VEGFR) and can prevent tumor growth. Other monoclonal antibodies, including siltuximab, abituzumab, trastuzumab, and cetuximab, bind to interleukin-6, integrin alpha-V, human epidermal growth factor receptor 2 (HER2), and epidermal growth factor receptor (EGFR), respectively [12-15]. Checkpoint inhibitors including nivolumab (anti-programmed cell death protein 1 (PD-1)), pembrolizumab (anti-PD-1), and ipilimumab (anti-cytotoxic T-lymphocyte-associated antigen-4 (CTLA-4)) are also tested in clinical trials for anti-tumor activity against CRPC [16,17]. While several of these immunotherapies are under evaluation in clinical trials, denosumab is the major monoclonal antibody approved by the FDA for metastatic bone lesions in CRPC [18].

The aim of this systematic review and meta-analysis is to assess the efficacy and safety of monoclonal antibodies alone or in combination with chemotherapy drugs in CRPC.

## Materials and methods

In conducting this systematic review and meta-analysis, we followed a prespecified protocol registered on the International Prospective Register of Systematic Reviews (PROSPERO) (registration number: CRD42021230102). The protocol was made according to the guidelines established by Cochrane [19] and PRISMA-P (Preferred Reporting Items for Systematic Review and Meta-Analysis Protocols) [20].

Search strategy

A literature search was performed on PubMed, Embase, Web of Science, Cochrane Library, and ClinicalTrials.gov with Medical Subject Heading (MeSH) and Emtree terms “monoclonal antibodies” and “castration-resistant prostate cancer.” The search was made from the inception of literature till March 20, 2021, by following the PICO framework (Appendix) [21].

Inclusion and exclusion criteria

We included all clinical trials that provided safety and efficacy data in clinical terms, i.e., objective response (OR), complete response (CR), partial response (PR), ≥50% decline in PSA, progression-free survival (PFS), overall survival (OS), and grade 3-4 adverse effects. We excluded all preclinical studies, case reports, meta-analyses, review articles, observation studies, and clinical studies irrelevant to the study question.

Study selection

Two researchers (WA and TAT) independently reviewed the articles identified through initial search and screened them based on inclusion and exclusion criteria. The differences were addressed by a third researcher (MAA).

Data extraction

Data were extracted by two authors (MS and MYA). The data were extracted for the characteristics of the study, baseline characteristics of participants, treatment drugs, efficacy measures, and toxicity (grade ≥ 3 adverse effects).

Risk of bias assessment

Two researchers (SR and SFB) assessed the risk of bias in randomized clinical trials (RCTs) selected for final inclusion by using the Risk of Bias 2 (RoB 2) tool for risk of bias assessment in RCTs [22]. The third researcher (MAA) addressed the differences.

Statistical analysis

The meta-analysis was performed using the “R” programming language. We used the “meta” package in R for our data analysis [23]. A random-effects model was used, irrespective of the heterogeneity, to keep our results consistent and applicable. All analyses used the DerSimonian-Laird estimator to calculate between-study variance. The risk ratios were pooled using the Mantel-Haenszel method. For studies with zero events in any of the arms, a continuity correction of 0.5 was used. Standard errors and other calculations were done using a 95% confidence interval. For pooling of the results, all the studies were included even if they have zero events in both arms. To estimate the heterogeneity, I^2^ was used.

## Results

**Figure 1:**
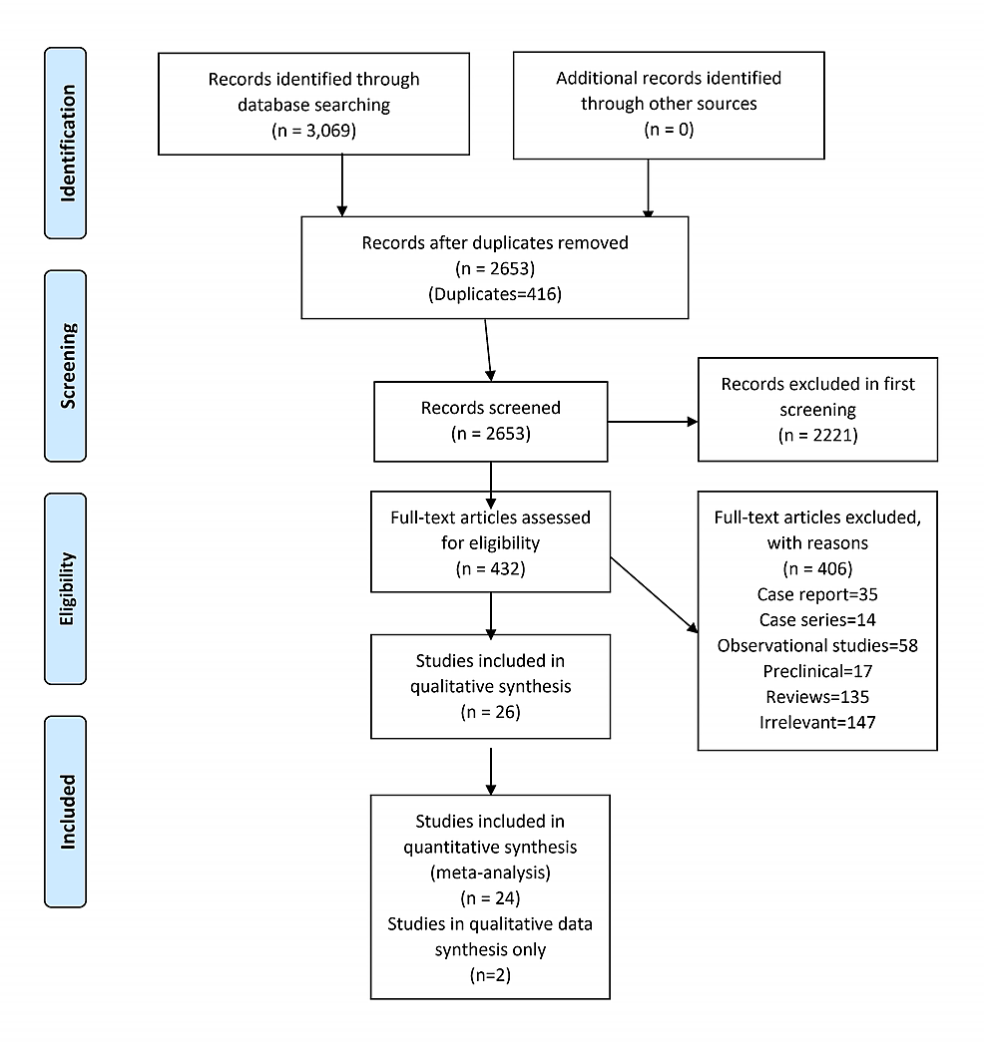
Flow chart of literature search.

A total of 3,069 articles were identified with 424 articles from PubMed, 2,427 articles from Embase, 49 articles from Web of Science, 60 articles from Cochrane, and 109 articles from ClinicalTrials.gov. These articles were analyzed by the researchers and 416 articles were removed as duplicates. A total of 2,221 articles were excluded in the first screening based on exclusion criteria. Full texts of 432 articles were reviewed. Eight RCTs (N = 6,227) [13,24-30] and 18 non-randomized clinical trials (NRCTs, N = 920) [10,15,31-41] were included based on prespecified inclusion criteria (Figure 1).

Risk of bias

**Figure 2:**
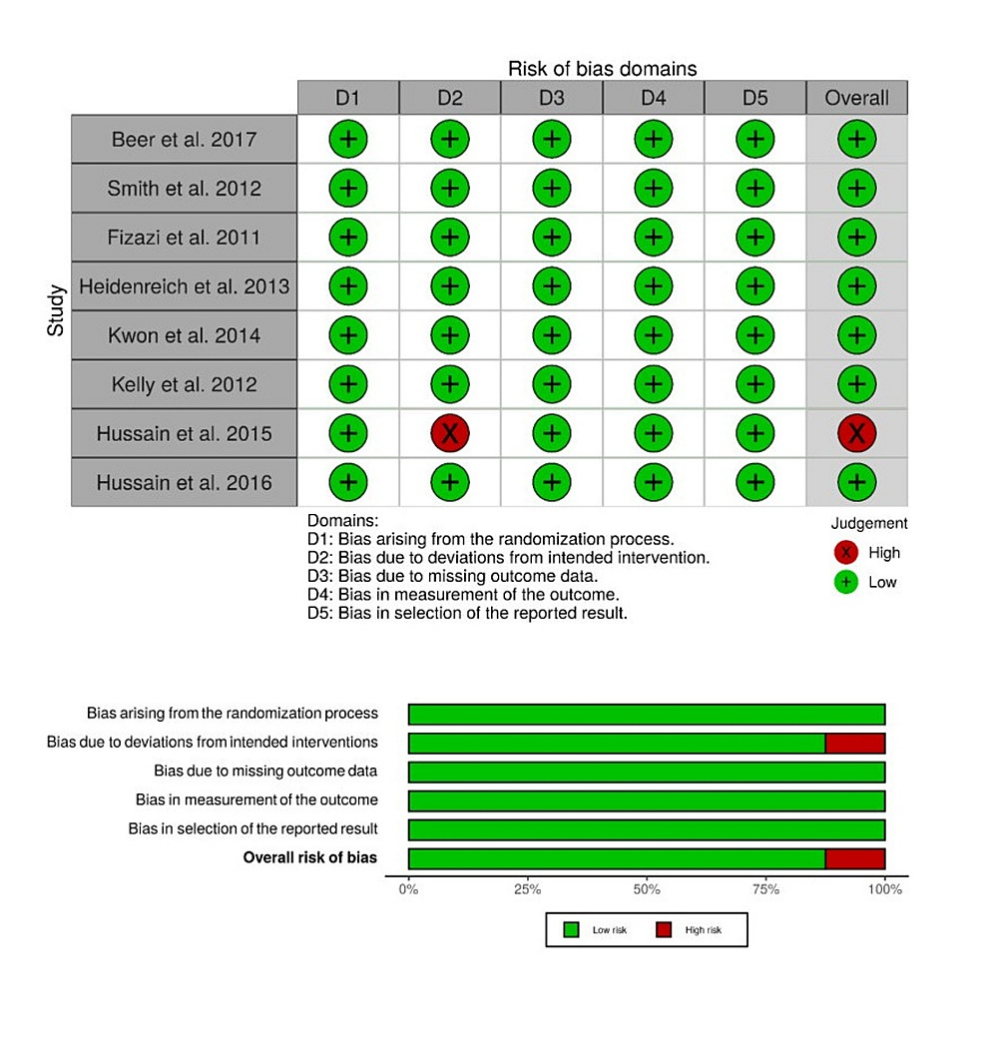
Risk of bias assessment with Risk of Bias 2 (RoB 2) tool. Studies [12,13,24-30].

The risk of bias was low in double-blinded RCTs except for open-label RCT conducted by Hussain et al. (2015) [30] (Figure 2).

Monoclonal antibodies vs. placebo

In six clinical trials (N = 4,194) [13,24-30], monoclonal antibodies were given to 2,225 participants while placebo was given to 1,969 participants. Standard of care (SOC) including luteinizing hormone-releasing hormone agonist/antagonist was given to 180 patients in the study by Hussain et al. [30]. The median ages of participants were ≥65 years in RCTs. Baseline characteristics of participants are given in Table 1.

**Table 1:** Baseline characteristics of trials. NCT = National Clinical Trial; ECOG = Eastern Cooperative Oncology Group; PSA = prostate-specific antigen; CTLA-4 = cytotoxic T-lymphocyte-associated antigen-4; RANKL = receptor activator of nuclear factor kappa-B ligand; M = mitoxantrone; P = prednisone; XRT= radiation therapy; SOC = standard of care; VEGFR = vascular endothelial growth factor receptor; EGFR = epidermal growth factor receptor; PD-1 = programmed cell death protein 1; IL-6 = interleukin 6; IGF = insulin-like growth factor; HER2 = human epidermal growth factor receptor 2; VEGF-A = vascular endothelial growth factor A.

Author, year	Phase	Trial NCT	Follow-up	N	Age	Treatment	ECOG score	Metastasis	Prior systemic therapy	Gleason score	Bone lesion	Median PSA
Randomized clinical trials												
Beer et al. (2017) [24]	III	NCT01057810	2-4 years	400	70 (44-91)	Ipilimumab (anti-CTLA-4, 10 mg/Kg)	0 = 75%, 1 = 25%	Bone = 78%	N/A	≤7 = 47%, ≥8 = 48%	Yes = 78%, no = 21%	41.2 (0.05-4,956)
				202	69 (42-92)	Placebo	0 = 75%, 1 = 25%	Bone = 79%	N/A	≤7 = 51%, ≥8 = 45%	Yes = 79%, no = 19%	49.5 (0.01-9,297)
Smith et al. (2012) [25]	III	NCT00286091	N/A	716	74·0 (67-80)	Denosumab (targets RANKL) (120 mg)	0 = 71%, 1 = 29%	Non-metastatic	N/A	≤7 = 60%, 8-10 = 30%	N/A	12·2 (4·7-27·5)
				716	74·0 (67-80)	Placebo	0 = 72%, 1 = 28%	Non-metastatic	N/A	≤7 = 56%, 8-10 = 33%	N/A	12·5 (4·9-28·5)
Fizazi et al. (2011) [26]	III	NCT00321620	12.2 month	950	71 (64-77)	Denosumab (targets RANKL)	0-1 = 93%	Visceral metastasis = 17%	Recent chemotherapy = 14%	2-6 = 18%, 7 = 29%, 8-10 = 41%	Skeletal event = 24%	58·5 (18·2-225·6)
			11.2 month	951	71 (66-77)	Zoledronic acid	0-1 = 93%	Visceral metastasis = 19%	Recent chemotherapy = 14%	2-6 = 19%, 7 = 29%, 8-10 = 43%	Skeletal event = 24%	60·0 (19·8-202·2)
Heidenreich et al. (2013) [27]	II	N/A	24 months	66	68 (41, 83)	Docetaxel + prednisone + intetumumab (integrin α-V, 10 mg/kg)	0 = 34, 1 = 30, 2 = 2	Metastatic cancer	57/66	<7 = 31, >7 = 22	N/A	N/A
			24 months	65	68 (46, 82)	Docetaxel + prednisone + placebo	0 = 31, 1 = 32, 2 = 2	Metastatic cancer	62/65	<7 = 26, >7 = 25	N/A	N/A
Kwon et al. (2014) [28]	III	NCT00861614	9.9 months	399	69·0 (47-86)	Ipilimumab group (anti-CTLA-4, 10 mg/kg)	0 = 168, 1 = 216, 2 = 3	Bone events <5 = 276, >5 = 103	N/A	<7 = 174, >7 = 192	Bone <5 = 276, >5 = 103	138·5 (0-4576)
			9.3 months	400	67·5 (45-86)	Placebo	0 = 170, 1 = 220	Bone events <5 = 253, >5 = 111	N/A	<7 = 190, >7 = 187	Bone <5 = 253, >5 = 111	176·5 (0-13768)
Kelly et al. (2012) [29]	III	N/A	8 cycles	524	68.8	Bevacizumab (anti-VEGF-A, 15 mg/kg) + docetaxel	0 = 57, 1 = 39, 2 = 4	Metastatic cancer	N/A	N/A	N/A	N/A
				526	69.3	Docetaxel	0 = 55, 1 = 40, 2 = 5	Metastatic cancer	N/A	N/A	N/A	N/A
Hussain et al. (2015) [30]	II	NCT00683475	N/A	66	65 (48-88)	Cixutumumab (anti-IGF, 6 mg/kg) + M + P	0 = 23, 1 = 38, 2 = 5	Metastatic cancer	Docetaxel-pretreated	N/A	N/A	133.45 (0.1-5530.0)
				66	68 (46-86)	Ramucirumab (VEGFR, 6 mg/kg) + M + P	0 = 19, 1 = 41, 2 =6	Metastatic cancer	Docetaxel-pretreated	N/A	N/A	107.30 (2.2-5826.4)
Hussain et al. (2016) [13]	II	NCT01360840	4.1 months	60	69.5 (54-84)	Abituzumab (anti-CD-51, 750 mg) and SOC	0 = 39, 1 = 18	Metastasis = 57	N/A	N/A	N/A	N/A
			4.2 months	60	71.0 (53-88)	Abituzumab 1,500 mg and SOC	0 = 34, 1 = 22	Metastasis = 59	N/A	N/A	N/A	N/A
			4.2 months	60	71.0 (46-88)	Placebo and SOC	0 = 32, 1 = 25	Metastasis = 59	N/A	N/A	N/A	N/A
Non-randomized clinical trials												
Vaishampayan et al. (2015) [31]	I	N/A	4 weeks	7	66-85	Anti-CD3 x anti-HER2 bispecific antibody	0-2 = 100%	Metastatic cancer	Hormones = 7, docetaxel = 1	6-9	Present	N/A
Picus et al. (2011) [33]	II	N/A	24 months	77	69 (48-88)	Estramustine, docetaxel, and bevacizumab ( anti-VEGF-A)	0-2 = 100%	Metastatic cancer	N/A	N/A	86%	123 ng/ml
Vaishampayan et al. (2014) [32]	II	N/A	24 months	30	67 (50-85)	Bevacizumab and satraplatin	N/A	Metastatic cancer	Docetaxel = 100%	6 = 6%, 7 = 26%, 8-10 = 65%	21 (68%)	180.7 ng/ml (4.7-1,432.8 ng/ml)
McNeel et al. (2018) [34]	II	N/A	N/A	26	73 (56-85)	Anti-tumor vaccine (+pembrolizumab-PD-1 inhibitor in 13)	<2	Metastatic cancer	Radiation, chemo, abiraterone, enzalutamide	<7 = 8%, 7 = 19%, 8 = 19%, 9 = 54%	N/A	24 (3-165)
Gross et al. (2017) [11]	Ib	NCT00574769	12 cycles + maintenance	43	65 (50-79)	Docetaxel, bevacizumab, and everolimus	N/A	Bone = 88%, nodes = 44%, viscera = 19%	Abiraterone = 26%, orteronel = 7%, enzalutamide = 5%	N/A	Bone metastasis = 88%	76.6 (0-1847)
Cathomas et al. (2012) [15]	II	NCT00728663	25.4 months	38	68 (45-82)	Docetaxel + cetuximab (EGFR inhibitor, 400 mg/m2)	N/A	Bone = 89%, node = 63%, visceral = 34%	1 regimen = 65%, 2 regimens = 26%, 3 regimens = 9% (docetaxel regimens)	N/A	Bone metastasis = 89%	212 ng/ml (4.4-8,898)
Batra et al. (2020) [35]	I	NCT00916123	N/A	15	69 (49-80)	Docetaxel + J591 (177Lu-J591)	0 = 40%, 1 = 53.3%, 2 = 6.7%	Bone = 93.3%, node = 60%, lung = 6.7%	Primary radiotherapy = 40%, salvage radiotherapy = 13.3%, prostatectomy = 46.7%	6 = 13.3%, 7 = 40%, 8-10 = 40%	Bone metastasis = 93.3%	84.32 ng/ml (17.2-776)
Madan et al. (2016) [36]	II	NCT00942578	47.5 months	63	65.6 (51-82)	Lenalidomide with bevacizumab, docetaxel, and prednisone	0 = 10, 1 = 50, 2 = 3	Bone = 24, bone + nodes = 27, bone + visceral = 7	N/A	≤6 = 4, 7 = 15, 8 = 15, 9 = 23, 10 = 6	Bone = 24	90.36 (0.14-3 520)
Slovin et al. (2013) [37]	I/II	NCT00323882	N/A	16	65 (53-76)	Ipilimumab (anti-CTLA-4, 10 mg/kg)	0 = 10, 1 = 6, 2 = 0	Metastatic cancer	6 (38%)	N/A	2.5 (1-12)	132 (13-2581)
				34	66 (50-83)	Ipilimumab = 10 mg/kg + XRT	0 = 9, 1 = 22, 2 = 0	Metastatic cancer	21 (62%)	N/A	8 (1-15)	120 (8-1314)
Barata et al. (2019) [38]	I/II	NCT01083368	N/A	21	64 (53-82)	Temsirolimus and bevacizumab	0 = 19%, 1 = 62%, 2 = 14%	Metastatic cancer	Docetaxel = 86%, mitoxantrone = 29%, ketoconazole = 24%, cabazitaxel = 10%, gemcitabine = 10%	<7 = 33%, >= 8 = 43%	21 (100%)	205.3 (11.1-1801.0)
Autio et al. (2020) [39]	I	NCT02265536	N/A	12	58-84	LY3022855	0 = 33%, 1 = 58.3%, 2 = 8%	Metastatic cancer	Chemotherapy = 42% Abiraterone acetate/enzalutamide = 100%	N/A	10/12 (83%)	N/A
Di Lorenzo et al. (2008) [40]	II	N/A	N/A	20	66 (49-73)	Bevacizumab + docetaxel	N/A	Metastatic cancer	Docetaxel = 100%, mitoxantrone = 100%, vinorelbine = 65%	<7 = 8, >7 = 12	Bone metastasis = 100%	260
Graff et al. (2020) [42]	II	NCT02312557	37 months	28	72 (61-90)	Pembrolizumab (anti-PD-1, 200mg) + enzalutamide	0 = 39%, 1 = 61%	Metastatic cancer	Docetaxel = 4, abiraterone = 10, enzalutamide = 28	<7 = 1, 7 = 9, >7 =1 4	Bone only = 13, bone and lymph nodes = 9	26.61 ng/ml (3.03-2502.75)
Francini et al. (2011) [43]	II	N/A	11.3 months	43	74 (58-82)	Docetaxel + bevacizumab + prednisone	0 = 20.9%, 1-2 = 79%	Metastatic cancer	w-epirubicin + w-docetaxel = 21 3-w, docetaxel + prednisone = 15, w-docetaxel + prednisone = 7	N/A	N/A	78 (47-374)
Ning et al. (2010) [44]	II	N/A	34 months	60	66 (44-79)	Docetaxel, bevacizumab, thalidomide, prednisone	0 = 13%, 1 = 80%, 2 = 7%	Metastatic cancer	N/A	<7 = 20 (33%), >8 = 39 (65%)	N/A	99 (0.9-4,399)
Hudes et al. (2013) [41]	I	N/A	N/A	39	66 (43, 82)	Docetaxel 75 mg/m2 + siltuximab (anti-IL-6, 6-12 mg/kg)	N/A	Metastatic cancer	N/A	8 (5,10)	N/A	57 (12, 1430)
Sharma et al. (2020) [16]	II	NCT02985957	11.9 months	45	69 (48-85)	Nivolumab (anti-PD-1, 1 mg/kg) + ipilimumab (anti-CTLA-4, 3 mg/kg)	0 = 26 (57.8%), 1 = 19 (42.2%)	M0 = 28 (62.2%), MI = 15 (33.3%)	Abiraterone = 66.7%, enzalutamide = 57.8%, bicalutamide = 55.6%, leuprolide = 60%, docetaxel = 11.1%	<7 = 35.5%, >7 = 60%	0 = 20%, <4 = 13.3%, >4 = 66.7%	59.5 ng/ml (93.3-1045)
			13.5 months	45	65 (46-84)	Nivolumab 1 mg/kg + ipilimumab (3 mg/kg)	0 = 25 (55.6) 1 = 20 (44.4%)	M0 = 22 (48.9%), MI = 20 (44.4%)	Abiraterone = 71.1%, enzalutamide = 62.2%, bicalutamide = 64.4%, leuprolide = 53.3%, docetaxel = 86.7%, cabazitaxel = 46.7%	7 or less = 42.2%, 8 or more = 51.1%	0 = 6.7%, <4 = 2.2%, >4 = 91.1%	158.9 ng/ml (1.8-1348.7)
Antonarakis et al. (2020) [17]	II	NCT02787005	9.5 months	133 PD-L1+	68 (48-85)	Pembrolizumab 200 mg	0 = 36%, 1 = 53.4%, 2 = 10%	Metastatic cancer	No. of previous chemotherapy regimens: 1 = 183 (71%), 2 or more = 75 (29%)	7 or less = 31.7%, 8 or more = 62%, unknown = 6.2%	Bone predominant = 59	115.5 (0.1-5000)
			7.9 months	66 PD-L1-	68 (53-84)							116.1 (1.0-3583.0)
			14.1 months	59	71 (53-90)							43.3 (0.1-2539.0)

Efficacy

**Figure 3:**
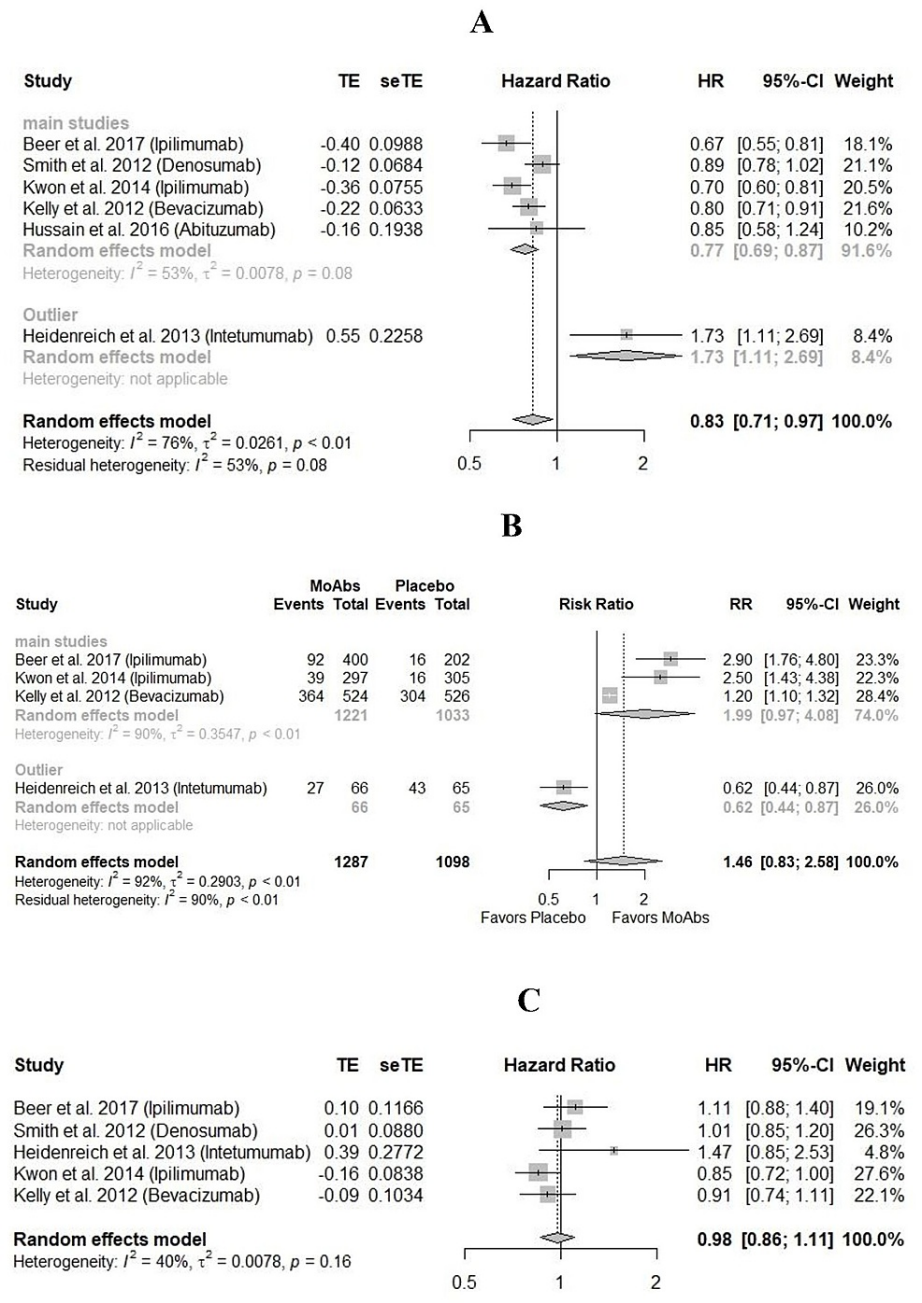
Comparison of efficacy in monoclonal antibodies vs. placebo. (A) Hazard ratio of progression-free survival. (B) Risk ratio of ≥50% decline in prostate-specific antigen (PSA). (C) Hazard ratio of overall survival [24-30]. MoAbs = monoclonal antibodies; TE = treatment effect; seTE: standard error of treatment effect.

In RCTs with ipilimumab, denosumab, bevacizumab, and abituzumab (N = 4,063), pooled hazard ratio (HR) of PFS was 0.77 (95% CI = 0.69-0.87, I^2 ^= 53) in favor of monoclonal antibodies as compared to placebo. HR of PFS for trial on intetumumab (N = 131) was 1.73 (95% CI = 1.11-2.69) in favor of placebo as compared to monoclonal antibodies (Figure 3A).

In RCTs with ipilimumab and bevacizumab (N = 2,254), the risk ratio (RR) of ≥50% decline in PSA was 1.99 (95% CI = 0.97-4.08, I^2 ^= 53%) in favor of monoclonal antibodies as compared to placebo. While in the RCT with intetumumab, RR of ≥50% decline in PSA was 0.62 (95% CI = 0.44-0.87) in favor of placebo as compared to monoclonal antibodies (Figure 3B).

In RCTs with ipilimumab, denosumab, bevacizumab, and intetumumab (N = 4014), HR of overall survival was similar in monoclonal antibodies groups vs. placebo, i.e., 0.98 (95% CI = 0.86-1.11, I^2 ^= 40%) (Figure 3C).

In RCT with denosumab (N = 1432), HRs of bone metastasis-free survival and first bone metastasis were statistically significant in favor of denosumab. HRs of bone metastasis-free survival and first bone metastasis were 0.85 (95% CI = 0.73-0.98) and 0.84 (95% CI = 0.71-0.98), respectively.

Safety

**Figure 4:**
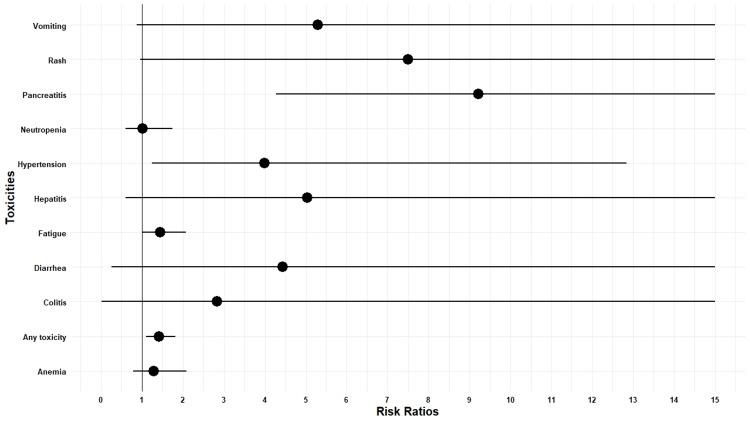
Plot of the risk ratio of ≥ grade 3 adverse events.

In RCTs, RRs of any ≥ grade 3 toxicity were 1.41 (CI = 1.10-1.82, I^2 ^= 92%) in favor of placebo as compared to monoclonal antibodies. RRs of ≥ grade 3 adverse events, i.e., vomiting, rash, pancreatitis, neutropenia, hypertension, hepatitis, fatigue, diarrhea, colitis, and anemia, were 5.30 (95% CI = 0.87-32.36, I^2 ^= 0), 7.50 (95% CI = 0.94-59.46, I^2 ^= 0), 9.21 (95% CI = 4.27-19.85), 1.01 (95% CI = 0.58-1.74, I^2 ^= 63.5%), 3.98 (95% CI = 1.23-12.84, I^2 ^= 19.2%), 5.02 (95% CI = 0.58-42.95, I^2 ^= 0), 1.44 (95% CI = 1.00-2.07, I^2 ^= 22.8%), 4.42 (95% CI = 0.25-75.69, I^2 ^= 81.1%), 2.82 (95% CI = 0.01-550.00, I^2 ^= 84.6%), and 1.28 (95% CI = 0.79-2.08, I^2 ^= 8.3%), respectively. Denosumab increased the incidence of ≥ grade 3 osteonecrosis of jaw in RCT 33/720 vs. 0/705 (Figure 4).

Denosumab vs. zoledronic acid

Fizazi et al. (2011) [26] compared denosumab vs. zoledronic acid for the treatment of CRPC (N = 1,904). HR of the first skeletal-related adverse event was 0.82 (95% CI = 0.71-0.95) in favor of denosumab as compared to zoledronic acid. The incidence of total skeletal-related events was 36% in the denosumab group vs. 41% in the zoledronic acid group. Radiation to bone was used in 19% of the people in the denosumab group vs. 21% in the zoledronic acid group. The incidence of adverse events was 97% each in both groups. Greater than or equal to grade 3 adverse events were 72% and 66% in denosumab and zoledronic acid groups, respectively. Osteonecrosis of the jaw was 1% in the zoledronic acid group vs. 2% in the denosumab group. Discontinuation of treatment due to adverse events was reported in 15% of participants in the zoledronic acid group and 17% in the denosumab group.

Cixutumumab vs. ramucirumab

Hussain et al. (2015) [30] compared cixutumumab vs. ramucirumab (N = 132). The median time to radiographic disease progression was 7.5 months (95% CI = 4.8-10.1) for patients on cixutumumab while it was 10.2 months (95% CI = 7.5-12.6) for patients on ramucirumab. Median OS was 10.8 months (95% CI = 6.5-13.0) for patients on cixutumumab while it was 13.0 months (95% CI = 9.5-16.0) for patients on ramucirumab. Decline >50% in PSA occurred in 18.5% of patients in the cixutumumab group and 21.4% of patients in the ramucirumab group. Among ≥ grade 3 adverse events, fatigue, diarrhea, dehydration, hypertension, neutropenia, and anemia were reported in 16.7% vs. 7.6%, 7.6% vs. 1.5%, 6.1% vs. 1.5%, 1.5% vs. 9.1%, 31.9% vs. 31.8%, and 3% vs. 10.6% of patients, respectively, in cixutumumab vs. ramucirumab groups.

Single-arm comparison of monoclonal antibody regimens

**Figure 5:**
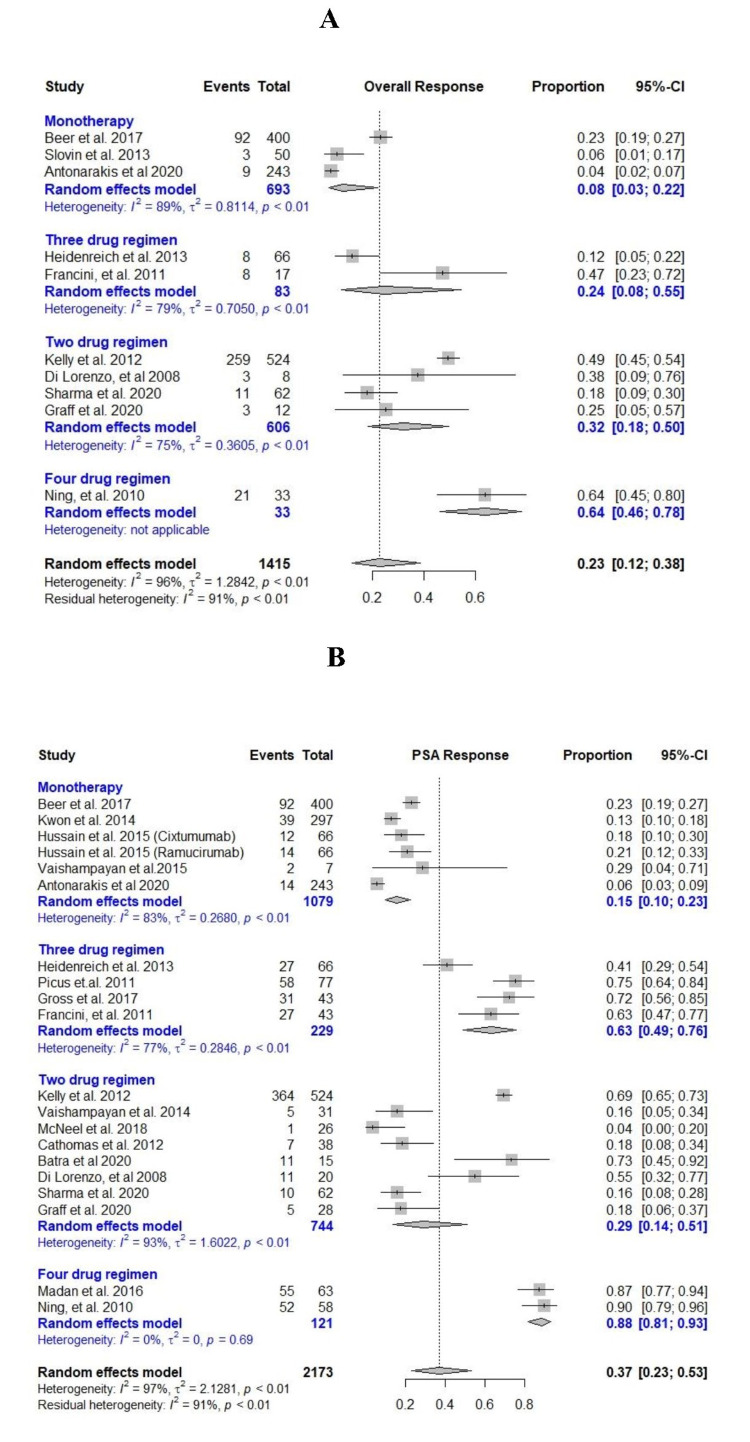
Meta-analysis of efficacy in single arms. (A) Pooled overall response. (B) Pooled >50% prostate-specific antigen (PSA) response [10,17,24-35].

Ipilimumab, cixutumumab, ramucirumab, anti-CD3 x anti-HER2 bispecific antibody, and pembrolizumab were used as monotherapy in clinical trials (N = 1,129) [17,24,28,30,31]. Pooled incidences of OR and >50% decline in PSA were 8% (95% CI = 0.03-0.22, I^2 ^= 89%) and 15% (95% CI = 0.1-0.23, I^2 ^= 83%), respectively. Individual study results and pooled results are given in Figure 5. Median OS and PFS were 7.4-19.6 months and 2.1 months, respectively (Table 2).

**Table 2:** Survival rates and ≥ grade 3 adverse events in early phase trials. PFS = progression-free survival; OS = overall survival.

Author	Median PFS (months)	Median OS (months)	Any ≥ grade 3	Diarrhea	Hypertension	Anemia	Neutropenia/lymphopenia	Colitis	Hepatitis	Fatigue	Rash	Vomiting
Monotherapy												
Vaishampayan et al. (2015) [31]	N/A	N/A	5/7	N/A	N/A	N/A	N/A	N/A	N/A	N/A	N/A	N/A
Antonarakis et al. (2020) [17]	2.1 (2.1-2.2)	9.6 (7.9-12.2)	27 (10%)	2 (<1%)	N/A	2 (<1%)	N/A	3 (1%)	1 (<1%)	3 (1%)	0	0
Slovin et al. (2013) [17]	N/A	17.4 (11.5-24.7)	N/A	4(8)	N/A	N/A	N/A	8 (16)	4 (8)	3 (6)	16 (32%)	3 (6%)
Autio et al. (2020) [39]	N/A	N/A	3	N/A	N/A	N/A	N/A	N/A	N/A	2	N/A	0
Two-drug regimens												
Vaishampayan et al. (2014) [32]	7.0 (4.7-8.5)	11.2 (9.1-16.4)	N/A	2/30	3/30	7/30	9/30	N/A	N/A	1/30	N/A	N/A
McNeel et al. (2018) [34]	N/A	N/A	N/A	1/26	N/A	N/A	N/A	N/A	1/26	1/26	N/A	N/A
Cathomas et al. (2012) [15]	2.8 (2.4-3.2)	13.3 (7.3-15.4)	N/A	1 (3%)	N/A	1 (3%)	3 (8%)	N/A	N/A	4 (11%)	2 (5%)	N/A
Batra et al. (2020) [35]	N/A	18.4 (16.13-NR)	N/A	N/A	N/A	N/A	11 (73.3%)	N/A	N/A	1 (6.66%)	N/A	N/A
Di Lorenzo et al. (2008) [40]	4 (2-6)	9 (4-12.5)	11/20 (55%)	N/A	N/A	1/20 (5%)	4 (20%)	N/A	N/A	N/A	N/A	2 (10%)
Sharma et al. (2020) [16]	5.5 (3.5-7.1) and 3.8 (2.1-5.1)	19 (11.5-NE) and 15.2 (8.4-NE)	43/85	8	N/A	N/A	N/A	N/A	N/A	N/A	N/A	N/A
Graff et al. (2020) [42]	3.8 (2.8-9.9)	22.2 (14.7-28.4)	19/28 (68%)	N/A	3 (10.7%)	1 (3.5%)	N/A	2 (7.1%)	N/A	1 (3.5%)	N/A	N/A
Barata et al. (2019) [38]	N/A	N/A	9 (43%)	N/A	N/A	N/A	N/A	N/A	N/A	5 (24%)	N/A	1 (5%)
Hudes et al. (2013) [41]	N/A	N/A	33/37	0	N/A	1	27/37 (73%)	N/A	N/A	N/A	N/A	N/A
Three-drug regimens												
Francini et al. (2011) [43]	N/A	N/A	16/43 (37.2%)	N/A	N/A	6 (13.9%)	8 (18.6%)	N/A	N/A	2 (4.6%)	N/A	N/A
Picus et al. (2011) [33]	9.2 (7.5-10.9)	24 (20.3-26.5	N/A	N/A	4/77 (5%)	N/A	53/77 (69%)	N/A	N/A	19 (24%)	N/A	N/A
Gross et al. (2017) [11]	8.9 (7.4-10.6)	21.9 (18.4-30.3)	N/A	N/A	8 (19%)	N/A	12 (28%)	N/A	N/A	3 (7%)	N/A	N/A
Four-drug regimens												
Ning et al. (2010) [44]	18.3	28.2	N/A	2/60 (3.33%)	7/60 (11.6%)	8/60 (13.3%)	60/60 (100%)	N/A	N/A	2/60 (3.33%)	N/A	N/A
Madan et al. (2016) [36]	18.2	24.6	N/A	6 (10%)	N/A	20 (32%)	61	N/A	N/A	6 (11%)	N/A	N/A

Bevacizumab + docetaxel, bevacizumab + satraplatin, anti-tumor vaccine + pembrolizumab, docetaxel + cetuximab, docetaxel + J591, bevacizumab + docetaxel, nivolumab + ipilimumab, and pembrolizumab + enzalutamide were two drug combination regimens used in clinical trials to treat CRPC (N = 744) [15,16,32,34,40,41]. Pooled incidences of OR and >50% decline in PSA were 32% (95% CI = 0.18-0.50, I^2 ^= 75%) and 29% (95% CI = 0.14-0.51, I^2 ^= 93%), respectively (Figure 5). Median OS and PFS were 9-19 months and 2.8-7 months, respectively (Table 2).

Intetumumab + docetaxel + prednisone, estramustine + docetaxel + bevacizumab, docetaxel + bevacizumab + everolimus, and docetaxel + bevacizumab + prednisone were the three-drug regimens used in clinical trials (N = 229) [10,27,33,40,43]. Pooled incidences of OR and >50% decline in PSA were 24% (95% CI = 0.08-0.55, I^2 ^= 79%) and 63% (95% CI = 0.49-0.76, I^2 ^= 77%), respectively (Figure 5). Median OS and PFS were 21.9-24 months and 9.2-8.9 months, respectively (Table 2).

Lenalidomide + bevacizumab + docetaxel + prednisone and docetaxel + bevacizumab + thalidomide + prednisone were the four-drug regimens used in clinical trials (N = 121) [36,44]. Pooled incidences of OR and >50% decline in PSA were 64% (95% CI = 0.46-0.78, I^2 ^= 0%) and 88% (95% CI = 0.81-0.93, I^2 ^= 0%), respectively (Figure 5). Median OS and PFS were 24.6-28.2 months and 18.2-18.3 months, respectively (Table 2).

Monoclonal antibodies with unfavorable results

Cixutumumab, figitumumab, carlumab, trastuzumab, LFA102, rilotumumab, and siltuximab did not show antitumor activity in early phase trials (Table 3) [12,14,45-50].

**Table 3:** Early phase trials on monoclonal antibodies with no anti-tumor activity. MoAb = monoclonal antibody; IGF-1R = insulin-like growth factor-1 receptor; CRPC = castration-resistant prostate cancer; PSA = prostate-specific antigen; CTLA-4 = cytotoxic T-lymphocyte-associated antigen-4; PD-1 = programmed cell death protein 1; MCP-1 = monocyte chemotactic protein-1; IL-6 = interleukin 6; HER2 = human epidermal growth factor receptor 2.

Author	Trial phase	Drug combination	Target of MoAB	Problem	Outcomes
McHugh et al. (2020) [45]	Phase I	Cixutumumab + temsirolimus	IGF-1R	Metastatic CRPC	The combination therapy had limited anti-tumor activity and a greater than expected toxicity
De Bono et al. (2014) [46]	Phase II	Figitumumab + docetaxel	IGF-1R	Metastatic CRPC	No significant PSA response. The combination not recommended by authors in Bono et al.
Boudadi et al. (2018) [48]	Phase II	Ipilimumab + nivolumab	CTLA-4, PD-1	Metastatic CRPC	Anti-tumor activity was only seen in patients with AR-V7 isoform of the androgen receptor. Tumor activity was not seen in other patients
Pienta et al. (2013) [47]	Phase II	Carlumab	MCP-1	Metastatic CRPC	Well tolerated but did not show anti-tumor activity as a single agent
Fizazi et al. (2012) [12]	Phase II	Siltuximab + mitoxantrone/prednisone	IL-6	Metastatic CRPC	The drug combination was well tolerated, improvement in outcomes was not demonstrated
Ziada et al. (2004) (NCT00003740) [14]	Phase II	Trastuzumab	HER2	CRPC	Well tolerated with no anti-tumor activity
Minami et al. (2020) (NCT01610050) [49]	Phase I	LFA102	Anti-prolactin receptor	Metastatic CRPC	Well tolerated with no anti-tumor activity
Ryan et al. (2013) (NCT00770848) [50]	Phase I/II	AMG 102 (rilotumumab)	Hepatocyte growth factor	Resistant CRPC	Well tolerated with no anti-tumor activity

Ongoing clinical trials and interim results of ongoing trials

Interim results of ongoing clinical trials on pembrolizumab, avelumab, atezolizumab, pasotuxizumab, and tremelimumab have shown promising results alone or in combination with chemotherapy [51-58]. Combinations are given in Table 4.

**Table 4:** Ongoing clinical trials and interim results of ongoing trials presented in conferences. NCT = National Clinical Trial; DPP4 = dipeptidyl peptidase 4; HER2 = human epidermal growth factor receptor 2; PD-1 = programmed cell death protein 1; PD-L1 = programmed death-ligand 1; CTLA-4 = cytotoxic T-lymphocyte-associated antigen-4; IL-23 = interleukin 23; CRPC = castration-resistant prostate cancer; PSA = prostate-specific antigen; PSMA = prostate-specific membrane antigen.

NCT/authors	No. of patients	Regimen	Target of antibody	Phase	Population	Outcome	Year of completion
Interim results of ongoing clinical trials							
Gurney et al. (2019) (NCT02861573) [51]	41	Pembrolizumab + olaparib	PD-1	Ib/II	Metastatic CRPC	PSA response 12%, well-tolerated	2025
Gurney et al. (2019) [51]	72	Pembrolizumab + docetaxel + prednisone	PD-1	Ib/II	Metastatic CRPC	PSA response 31%, well-tolerated	2025
Gurney et al. (2019) [51]	69	Pembrolizumab + enzalutamide	PD-1	Ib/II	Metastatic CRPC	PSA response 27%, well-tolerated	2025
Bryce et al. (2020) (NCT03409458) [52]	14	Avelumab + PT-112	PD-L1	I/II	Metastatic CRPC	Well tolerated with evidence of efficacy, PSA response 21%	2021
Aggarwal et al. (2020) (NCT03910660) [53]	6	BXCL701 (DPP4 inhibitor) + pembrolizumab	PD-1	Ib	Metastatic CRPC	Well tolerated	2022
Patel et al. (2020) (NCT03406858) [54]	33	Pembrolizumab + HER2 bi-armed activated T cells	PD-1	II	Metastatic CRPC	PSA response 2/6 patients, well-tolerated	2021
Dorff et al. (2020) (NCT03024216) [55]	37	Atezolizumab + sipuleucel-T	PD-L1	I	Metastatic CRPC	Well tolerated with clinical activity	2025
Agarwal et al. (2020) (NCT03170960) [56]	44	Cabozantinib + atezolizumab	PD-L1	Ib	Metastatic CRPC	Well tolerated with clinical activity	2021
Hummel et al. (2021) (NCT01723475) [57]	47	Pasotuxizumab, PSMA bispecific T-cell engager monotherapy	PSMA	I	Metastatic CRPC	Well tolerated with clinical activity	2018
Hotte et al. (2019) (NCT02788773) [58]	52	Durvalumab with or without tremelimumab	CTLA-4 + PD-L1	II	Metastatic CRPC	No activity with durvalumab only, clinical activity reported with combination therapy	2020
Ongoing clinical trials							
NCT03815942	23	Nivolumab + ChAdOx1-MVA 5T4 vaccine	Anti-PD-1	I/II	CRPC	Efficacy and safety (active, not recruiting)	2021
NCT04458311	55	Tildrakizumab + abiraterone acetate	Anti-IL-23	I/II	Metastatic CRPC	Efficacy and safety (recruiting)	2024
NCT03204812	27	Durvalumab plus tremelimumab	Anti-PD-L1 and anti-CTLA-4	II	Metastatic CRPC	Efficacy and safety (active, not recruiting)	2021
NCT04336943	30	Durvalumab + olaparib	Anti-PD-L1	II	Biochemically recurrent prostate cancer	Efficacy and safety (recruiting)	2024
NCT03910660	40	Talabostat mesylate + pembrolizumab	Anti-PD-1	I/II	Metastatic CRPC	Efficacy and safety (active, not recruiting)	2022
NCT04071236	24	Radium Ra 223 + peposertib + avelumab	Anti-PD-L1	I/II	Advanced metastatic CRPC	Efficacy and safety (recruiting)	2023
NCT04104893	30	Pembrolizumab	Anti-PD-1	II	Metastatic CRPC characterized by a mismatch repair deficiency or biallelic CDK12 inactivation	Efficacy and safety (recruiting)	2023
NCT02703623	198	Abiraterone acetate, apalutamide, prednisone +/-ipilimumab	Anti-CTLA-4	II	Metastatic CRPC	Efficacy and safety (active, not recruiting)	2022
NCT04159896	49	ESK981 + nivolumab	Anti-PD-1	II	Metastatic CRPC	Efficacy and safety (recruiting)	2022
NCT03367819	134	Isatuximab + cemiplimab	Anti-CD-38 and Anti-PD-1	I/II	Metastatic CRPC	Efficacy and safety (active, not recruiting)	2021
NCT03805594	43	Lutetium Lu 177-PSMA-617 + pembrolizumab	Anti-PSMA + anti-PD-1	I	Metastatic CRPC	Efficacy and safety (recruiting)	2022
NCT02499835	66	Vaccine therapy + pembrolizumab	Anti-PD-1	I/II	Metastatic CRPC	Efficacy and safety (active, not recruiting)	2021
NCT04471974	54	Pembrolizumab + ZEN-3694 + enzalutamide	Anti-PD-1	II	Metastatic CRPC	Efficacy and safety (recruiting)	2025
NCT04592237	120	Cetrelimab + cabazitaxel + carboplatin + niraparib	Anti-PD-1	II	Aggressive prostate cancer	Efficacy and safety (recruiting)	2025
NCT02312557	58	Pembrolizumab + enzalutamide	Anti-PD-1	II	Metastatic CRPC	Efficacy and safety (active, not recruiting)	2022
NCT03217747	184	PF-04518600 + avelumab + utomilumab	Anti-OX40, anti-PDL1, and anti-CD137	I/II	Patients with advanced malignancies	Efficacy and safety (recruiting)	2023
NCT02601014	15	Ipilimumab + nivolumab	Anti-CTLA-4 and anti-PD-1	II	AR-V7-expressing metastatic CRPC	Efficacy and safety (active, not recruiting)	2022
NCT04068896	90	NGM120	GFRAL antagonist blocking GDF15	I	Metastatic CRPC	Efficacy and safety (recruiting)	2021
NCT03849469	242	Pembrolizumab + XmAb22841	Anti-PD-1 + anti-CTLA-4	I	Selected advanced solid tumors (DUET-4)	Efficacy and safety (recruiting)	2027
NCT03517488	154	XmAb20717	Anti-PD-1/anti-CTLA-4	I	Advanced solid tumors	Efficacy and safety (recruiting)	2021
NCT03454451	378	CPI-006 + pembrolizumab	Anti CD73 + anti-PD-1	I	Metastatic CRPC	Efficacy and safety (recruiting)	2023
NCT03330405	216	Avelumab + talazoparib	Anti-PD-L1	II	CRPC	Efficacy and safety (active, not recruiting)	2021
NCT04423029	260	Nivolumab + DF6002	Anti-PD-1	I/II	Metastatic solid tumors	Efficacy and safety (recruiting)	2024
NCT03207867	376	PDR001 + NIR178	Anti-PD-1	II	CRPC, solid tumors, and lymphoma	Efficacy and safety (recruiting)	2021
NCT03983954	45	Naptumomab + durvalumab	Anti-5T4 and anti-PD-L1	I	Solid tumor that is metastatic/advanced	Efficacy and safety (recruiting)	2022
NCT03970382	148	Nivolumab	Anti-PD-1	I	Locally advanced or metastatic solid tumors	Efficacy and safety (recruiting)	2024

## Discussion

Docetaxel is the most used chemotherapy-based treatment for metastatic CRPC as docetaxel improved OS, PFS, and PSA levels in RCT [59]. Among non-chemotherapy drugs, alpharadin, abiraterone, radium-223 dichloride, etc., showed improvement in survival rates with anti-tumor activity [60]. Among immunotherapies, sipuleucel-T extended OS without improving PFS [61]. However, these therapies are not curative, responses are rarely durable, and are poorly tolerated by some patients. Additional treatment options are needed for better outcomes. In RCTs, majorly monoclonal antibodies were used in combination with docetaxel or in patients refractory to docetaxel therapy. According to the pooled results, monoclonal antibodies improved PFS and PSA response as compared to placebo.

Checkpoint inhibitors, including PD-1, programmed death-ligand 1 (PD-L1), and CTLA-4 inhibitors, have shown efficacy in urothelial and other solid tumors [62-65]. However, the microenvironment of prostate cancer is more immunosuppressive as compared to other tumors [66,67]. Ipilimumab (CTLA-4 inhibitor) improved PFS and PSA levels in both trials, including docetaxel pre-treated and treatment naïve patients. It was well tolerated in both trials. OS was not prolonged on normal follow-up. However, long-term follow-up of five years showed better OS in the ipilimumab group as compared to placebo [68]. More trials are now conducted on combination therapy of ipilimumab. In the trial conducted by Boudadi et al. (2018) [48], 1 mg/kg of ipilimumab was used with nivolumab and anti-tumor activity was only reported in a small group of patients. However, according to the preliminary results of a trial by Sharma et al. (2020), 3 mg of ipilimumab with nivolumab showed anti-tumor activity in all subsets of patients and a large-scale phase II trial is in progress on ipilimumab + nivolumab in metastatic CRPC patients [16]. Another RCT is in progress to assess the efficacy and safety of ipilimumab in combination with abiraterone acetate, apalutamide, and prednisone. Ongoing clinical trials are also testing nivolumab in combination with ChAdOx1-MVA 5T4 vaccine, ESK981 (Pan-VEGFR/Tie2 tyrosine kinase inhibitor), and DF6002 (binds interleukin 12 (IL-12) receptor).

In a multicohort phase II trial by Antonarakis et al. (2020), pembrolizumab showed anti-tumor activity in docetaxel pretreated patients and the observed survival estimates are promising [17]. Although 5% of the patients showed OR, the response was durable. Pembrolizumab monotherapy was well tolerated, and no unexpected toxicities were reported. A combination of pembrolizumab with olaparib, enzalutamide, and docetaxel is tested in KEYNOTE-365, and the early results have shown anti-tumor activity of these combinations and are well tolerated [51]. According to the results of a phase II trial by Graff et al. (2020), pembrolizumab addition to enzalutamide showed anti-tumor activity in patients refractory to enzalutamide alone, and the response was durable [42]. Another trial was conducted on the addition of pembrolizumab to the anti-tumor DNA vaccine. The addition of pembrolizumab showed better results in terms of PSA decline, OR, and CD-8+ T cell infiltration into tumor lesions as compared to vaccination alone. More trials are in progress to assess the efficacy and safety of pembrolizumab in combination with dipeptidyl peptidase 4 (DPP4) inhibitor BXCL701, HER2 bi-armed activated T cells, talabostat mesylate, lutetium lu 177-PSMA-617, vaccine therapy, ZEN-3694 + enzalutamide, enzalutamide, XmAb22841, and CPI-006 (Table 4). Avelumab, atezolizumab, tremelimumab, cemiplimab, cetrelimab, XmAb20717, PDR001, and durvalumab are other checkpoint inhibitors that are getting tested alone and in combination therapy for the treatment of CRPC.

The anti-angiogenic drug, bevacizumab, also improved PFS and PSA levels without any improvement in OS. Bevacizumab was also tested in combination regimens. Among the combination regimens, the four-drug regimen of bevacizumab with docetaxel + thalidomide + prednisone and lenalidomide + docetaxel + prednisone showed the best efficacy outcomes, and toxicities were manageable (Figure 5 and Table 2). Early anti-tumor activity was reported with the addition of thalidomide and bevacizumab to docetaxel as compared to docetaxel alone. Bevacizumab in combination with satraplatin has shown promising results in early phase trials in docetaxel refractory patients. The addition of everolimus (mammalian target of rapamycin (mTOR) inhibitor) to docetaxel + bevacizumab did not show better outcomes as compared to docetaxel + bevacizumab in the early-phase trial.

Abituzumab improved the progression of the disease, but the results were not statistically significant. In our analysis, the trial with intetumumab was the outlier and intetumumab caused worsening in PFS or PSA levels. However, intetumumab did not increase adverse events as compared to placebo. Intetumumab might have interacted with docetaxel, resulting in lower efficacy.

Lack of improvement in OS despite changes in PFS and PSA levels might be due to the unique response of CRPC to these drugs. Also, the patients with metastatic CRPC are generally older than patients with other types of cancer, e.g., breast cancer and lung cancer, and comparatively more patients have bone metastasis [69-71]. Other possible explanations can be the unique mechanism of action of these drugs or flaws in trial designs. These drugs might show some improvement in OS if followed for longer durations. Further studies should be conducted on how to utilize the anti-tumor activity of these monoclonal antibodies.

Denosumab targets receptor activator of nuclear factor kappa-B ligand (RANKL) and is an anti-bone resorptive agent. It delayed skeletal-related adverse events as compared to zoledronic acid in patients with CRPC with bone metastasis in RCT. Zoledronic acid was proved better than a placebo in an RCT [72]. However, increased incidence of osteonecrosis of the jaw was associated with denosumab as compared to zoledronic acid. A meta-analysis showed similar results for denosumab in the prevention of skeletal-related adverse events as compared to zoledronic acid [73]. Moreover, an RCT by Smith et al. (2012) tested denosumab for the prevention of bone metastasis [25]. Denosumab significantly improved bone metastasis-free survival and time to first bone metastasis as compared to placebo. The major adverse event observed in the denosumab group was the osteonecrosis of the jawbone.

In a non-comparative randomized study, cixutumumab (IGF-1R inhibitor) and ramucirumab (VEGFR inhibitor) were used with mitoxantrone-prednisone. PFS in the cixutumumab group was similar to the projected value, while ramucirumab showed better PFS as compared to the projected value (6.7 months vs. 3.9 months). The incidence of adverse events was similar to expectations. Ramucirumab has shown improvement in OS in RCTs on other solid tumors [74]. Another trial by McHugh et al. (2020) has also shown no activity of cixutumumab with temsirolimus [45].

Among monoclonal antibodies, PD-1 inhibitors, PD-L1 inhibitors, and CTLA-4 inhibitors have the potential to become the drugs of the future for patients with prostate cancer. More multicenter randomized clinical trials should focus on finding the efficacy and appropriate combination of these medications. However, the role of monoclonal antibodies in prostate cancer is still debated.

## Conclusions

Monoclonal antibodies were well tolerated and showed better outcomes in terms of PFS and >50% decline in PSA levels compared to placebo. However, OS was only improved with ipilimumab as compared to placebo on long-term follow-up of five years. Denosumab delayed skeletal-related adverse events as compared to zoledronic acid in CRPC with bone metastasis. Denosumab also delayed bone metastasis as compared to placebo in patients with metastatic CRPC. Pembrolizumab, avelumab, atezolizumab, pasotuxizumab, and tremelimumab have shown promising results in the early phase trials. More multicenter, double-blind clinical trials may be needed to confirm these results.

## Appendices

**Table 5:** Keywords and search strings.

P	I	C	O	S
"Prostatic Neoplasms, Castration-Resistant"[Mesh]	"Antibodies, Monoclonal"[Mesh]			
Prostatic Neoplasms, Castration-Resistant Castration-Resistant Prostatic Neoplasm Prostatic Neoplasms, Castration-Resistant Androgen-Insensitive Prostatic Neoplasms Androgen Insensitive Prostatic Neoplasms Androgen-Resistant Prostatic Neoplasms Androgen Resistant Prostatic Neoplasms Prostatic Neoplasms, Hormone Refractory Hormone Refractory Prostatic Neoplasms Prostatic Neoplasms, Androgen-Independent Neoplasm, Androgen-Independent Prostatic Prostatic Neoplasm, Androgen-Independent Prostatic Neoplasms, Androgen Independent Prostatic Neoplasms, Androgen-Insensitive Androgen-Insensitive Prostatic Neoplasm Prostatic Neoplasms, Androgen Insensitive Prostatic Neoplasms, Androgen-Resistant Androgen-Resistant Prostatic Neoplasm Prostatic Neoplasm, Androgen-Resistant Prostatic Neoplasms, Androgen Resistant Androgen-Independent Prostatic Neoplasms Androgen Independent Prostatic Neoplasms Castration-Resistant Prostatic Neoplasms Castration-Resistant Prostatic Neoplasms Cancers, Castration-Resistant Prostatic Androgen-Insensitive Prostatic Cancer Androgen Insensitive Prostatic Cancer Androgen-Resistant Prostatic Cancer Androgen Resistant Prostatic Cancer Prostatic Cancer, Hormone Refractory Prostatic Cancer, Androgen-Independent Androgen-Independent Prostatic Cancers Prostatic Cancer, Androgen Independent Prostatic Cancers, Androgen-Independent Prostatic Cancer, Androgen-Insensitive Androgen-Insensitive Prostatic Cancers Cancer, Androgen-Insensitive Prostatic Cancers, Androgen-Insensitive Prostatic Prostatic Cancer, Androgen Insensitive Prostatic Cancers, Androgen-Insensitive Prostatic Cancer, Androgen-Resistant Androgen-Resistant Prostatic Cancers Cancer, Androgen-Resistant Prostatic Cancers, Androgen-Resistant Prostatic Prostatic Cancer, Androgen Resistant	Monoclonal Antibodies, Monoclonal Antibody, Antibody, Monoclonal			
PubMed search string: (((((((((((((((((((((((((((((((((((((((((((((("Prostatic Neoplasms, Castration-Resistant"[Mesh]) OR (Prostatic Neoplasms, Castration-Resistant)) OR (Castration-Resistant Prostatic Neoplasm)) OR (Prostatic Neoplasms, Castration Resistant)) OR (Androgen-Insensitive Prostatic Neoplasms)) OR (Androgen Insensitive Prostatic Neoplasms)) OR (Androgen-Resistant Prostatic Neoplasms)) OR (Androgen Resistant Prostatic Neoplasms)) OR (Prostatic Neoplasms, Hormone Refractory)) OR (Hormone Refractory Prostatic Neoplasms)) OR (Prostatic Neoplasms, Androgen-Independent)) OR (Neoplasm, Androgen-Independent Prostatic)) OR (Prostatic Neoplasm, Androgen-Independent)) OR (Prostatic Neoplasms, Androgen Independent)) OR (Prostatic Neoplasms, Androgen-Insensitive)) OR (Androgen-Insensitive Prostatic Neoplasm)) OR (Prostatic Neoplasms, Androgen Insensitive)) OR (Prostatic Neoplasms, Androgen-Resistant)) OR (Androgen-Resistant Prostatic Neoplasm)) OR (Prostatic Neoplasm, Androgen-Resistant)) OR (Prostatic Neoplasms, Androgen Resistant)) OR (Androgen-Independent Prostatic Neoplasms)) OR (Androgen Independent Prostatic Neoplasms)) OR (Castration-Resistant Prostatic Neoplasms)) OR (Castration Resistant Prostatic Neoplasms)) OR (Cancers, Castration-Resistant Prostatic)) OR (Androgen-Insensitive Prostatic Cancer)) OR (Androgen Insensitive Prostatic Cancer)) OR (Androgen-Resistant Prostatic Cancer)) OR (Androgen Resistant Prostatic Cancer)) OR (Prostatic Cancer, Hormone Refractory)) OR (Prostatic Cancer, Androgen-Independent)) OR (Androgen-Independent Prostatic Cancers)) OR (Prostatic Cancer, Androgen Independent)) OR (Prostatic Cancers, Androgen-Independent)) OR (Prostatic Cancer, Androgen-Insensitive)) OR (Androgen-Insensitive Prostatic Cancers)) OR (Cancer, Androgen-Insensitive Prostatic)) OR (Cancers, Androgen-Insensitive Prostatic)) OR (Prostatic Cancer, Androgen Insensitive)) OR (Prostatic Cancers, Androgen-Insensitive)) OR (Prostatic Cancer, Androgen-Resistant)) OR (Androgen-Resistant Prostatic Cancers)) OR (Cancer, Androgen-Resistant Prostatic)) OR (Cancers, Androgen-Resistant Prostatic)) OR (Prostatic Cancer, Androgen Resistant)) AND ((((("Antibodies, Monoclonal"[Mesh]) OR (Monoclonal Antibodies)) OR (Monoclonal Antibody)) OR (Antibody, Monoclonal)) AND (((((((((((("Prostatic Neoplasms"[Mesh]) OR (Prostatic Neoplasms)) OR (Neoplasms, Prostate)) OR (Prostate Neoplasm)) OR (Neoplasms, Prostatic)) OR (Prostatic Neoplasm)) OR (Prostate Cancer)) OR (Prostate Cancers)) OR (Cancer of the Prostate)) OR (Prostatic Cancer)) OR (Prostatic Cancers)) OR (Cancer of Prostate))) = 424				
Embase search string: ('castration resistant prostate cancer'/exp OR 'crpc (castration resistant prostate cancer)' OR 'castrate resistant prostate cancer' OR 'castration resistant prostate cancer' OR 'castration-resistant pc' OR 'castration-resistant pca' OR 'castration-resistant prostatic neoplasms' OR 'hormone refractory prostate cancer' OR 'prostatic neoplasms, castration-resistant') AND ('monoclonal antibody'/exp OR 'antibodies, monoclonal' OR 'antibodies, monoclonal, humanized' OR 'antibodies, monoclonal, murine derived' OR 'antibodies, monoclonal, murine-derived' OR 'antibody, monoclonal' OR 'clonal antibody' OR 'hybridoma antibody' OR 'monoclonal antibodies' OR 'monoclonal antibody') = 2,427				
Web of Science: with keywords mentioned above = 49				
Cochrane: with keywords mentioned above = 60				
ClinicalTrials.gov: prostate cancer + monoclonal antibodies = 109				
Total = 2,960				
